# On-the-fly learning for visual search of large-scale image and video datasets

**DOI:** 10.1007/s13735-015-0077-0

**Published:** 2015-03-22

**Authors:** Ken Chatfield, Relja Arandjelović, Omkar Parkhi, Andrew Zisserman

**Affiliations:** Visual Geometry Group, Department of Engineering Science, University of Oxford, Oxford, UK

**Keywords:** Object category retrieval and recognition, Object instance retrieval, Face retrieval, On-the-fly, Convolutional neural networks

## Abstract

The objective of this work is to visually search large-scale video datasets for semantic entities specified by a text query. The paradigm we explore is constructing visual models for such semantic entities *on-the-fly*, i.e. at run time, by using an image search engine to source visual training data for the text query. The approach combines fast and accurate learning and retrieval, and enables videos to be returned within seconds of specifying a query. We describe three classes of queries, each with its associated visual search method: object instances (using a bag of visual words approach for matching); object categories (using a discriminative classifier for ranking key frames); and faces (using a discriminative classifier for ranking face tracks). We discuss the features suitable for each class of query, for example Fisher vectors or features derived from convolutional neural networks (CNNs), and how these choices impact on the trade-off between three important performance measures for a real-time system of this kind, namely: (1) accuracy, (2) memory footprint, and (3) speed. We also discuss and compare a number of important implementation issues, such as how to remove ‘outliers’ in the downloaded images efficiently, and how to best obtain a single descriptor for a face track. We also sketch the architecture of the real-time on-the-fly system. Quantitative results are given on a number of large-scale image and video benchmarks (e.g.  TRECVID INS, MIRFLICKR-1M), and we further demonstrate the performance and real-world applicability of our methods over a dataset sourced from 10,000 h of unedited footage from BBC News, comprising 5M+ key frames.

## Introduction

One of the dreams of large-scale image search is to be able to retrieve images based on their visual content with the same ease, speed and in particular accuracy, as a Google search of the Web. Achieving this objective has been hampered for the most part by a semantic gap between the target category (e.g. ‘Gothic Cathedrals’) and the image features available to represent the data. In this paper we explore a method for bridging the gap by learning from readily available images downloaded from the Web with standard image search engines (such as Google Image search). In this manner the semantic gap is obviated for several types of query (see below), allowing powerful visual models to be constructed on the basis of freeform text queries.

A second aspect of the objective is to be able to achieve results immediately, and this requires that learning of a category occurs ‘on-the-fly’ at search time, as well as the scalable and immediate search of large-scale datasets. Putting the two together allows a user to start with a text query, learn a visual model for the specified category and then search an unannotated dataset on its visual content with results retrieved within seconds.

Computer vision researchers saw the potential of image search engines as soon as they were introduced [4, 16, 17, 31, 33, 45]. Early papers were concerned with improving the quality of the returned images, for example by reranking based on visual consistency to promote the target class. However, due to click-through crowd sourcing, the quality of the images is now extremely high over a vast variation of queries, to the extent that for most queries the first 100 or so top-ranking images are for the most part free of non-class images. The problem of visual polysemy still remains [45], (e.g. ‘Jaguar’ the car versus ‘jaguar’ the cat), but to an extent this can be avoided by more specific search queries (‘Jaguar car’ or ‘jaguar cat’) or by employing the clusters automatically provided by the search engines.

Learning on-the-fly from a reservoir of annotated images (the Web or proprietary datasets) has been investigated by a number of groups, including [1, 5, 8, 9, 18, 34, 39, 48, 52]. Such learning is in contrast to the more conventional approach of using hand-curated collections of positive and negative training images, such as PASCAL VOC [15] or ImageNet [12], where the set of categories is preset. On-the-fly learning offers a way to overcome the ‘closed world’ problem in computer vision, where object category recognition systems are restricted to only these pre-defined categories. There are applications to searching video archives, such as those of the BBC, and to searching personal image and video collections [29, 30], since both archives and personal collections have only sparse textual annotations at best.

This paper explores learning on-the-fly starting from a text query for immediate retrieval from large-scale video datasets, although the methods are equally applicable to image datasets. In the following we describe approaches suitable for learning and retrieving three classes of queries from downloaded images, each using a different technology. First, in Sect. 2, we consider *object and scene instances* such as specific places, scenes or objects, e.g. the White House, the Mona Lisa painting and an HSBC logo. We compare a number of methods of using an image set for retrieval and also describe a method for learning from noisy labels, namely, the query object is inferred from the downloaded image set by very efficiently identifying and eliminating any ‘outlier’ images. Second, in Sect. 3, we consider *object and scene categories* such as cars, crowds, and forests. The difference with the instance case is that a discriminative approach is used, requiring negative training images in addition to the ‘positive’ downloaded training images for the target category. Third, Sect. 4 describes an approach for *face* retrieval, for example to search for a particular person, such as President Obama. This also uses discriminative learning, but applied to tracked faces, rather than to individual images/key frames used in the instance and category search.

We then describe an architecture that allows these three approaches to be employed in an on-the-fly manner (Sect. 5), where text-to-image search using e.g. Google Image Search as the source of training images allows videos to be retrieved from large-scale datasets in a matter of seconds. We give particular attention to the retrieval performance/memory/speed trade-off inherent to such a system.

Throughout the paper we provide quantitative evaluations on a number of standard large-scale video datasets, including MIRFLICKR-1M [19, 20] and TRECVID [36, 37], and qualitative examples on a video dataset provided by the BBC of News broadcasts. This covers all news programmes broadcast over all BBC channels from 6 pm until midnight from 2007 to 2012. It consists of 10,132 h of footage from 17,401 different programmes, and is represented by 5,297,206 key frames.

This submission builds on a number of our previous conference papers [1, 8, 9, 39].

## Object instance retrieval

Here, we describe the first search modality, namely searching for specific object instances, such as specific buildings, logos and paintings in a large-scale image database. The aim of this specific object search is to instantaneously find key frames that contain the query object in the video dataset despite changes in scale, viewpoint, cropping and partial occlusion. This competence is useful in a number of settings, for example media production teams are interested in searching internal databases for images or video footage to accompany news reports and newspaper articles.

Current systems, for example Google Goggles, concentrate on querying using a single view of an object, e.g. a photo a user takes with his mobile phone, to answer the question ‘what is this?’. Here, we consider the somewhat converse problem of finding *all* images of an object given that the user knows what he is looking for; so the input modality is text, not an image. The problem is tackled in two stages: textual Google Image search is used to gather crowd-sourced images of the textual query, which are then in turn used to issue a visual query to search our image database.

A question arises as to how to use multiple query images (the query set), as current systems only issue a single visual query at a time. We propose three methods for doing this: method (i) uses the query set jointly to issue a single query (*early fusion*), while methods (ii) and (iii) issue a query for each image in the query set and combine the retrieved results (*late fusion*). The three methods are described next.

### Retrieval methods

*(i) Joint concatenated query (Joint-Concat)* Similar to the average query expansion method of [11], all descriptors from all images in the query set are concatenated into a single set of query descriptors, which is then used to rank database images using an existing single-query retrieval method.

*(ii) Maximum of multiple queries (MQ-Max)* A query is issued for each image in the query set independently and retrieved ranked lists are combined by scoring each retrieved image by the maximum of the individual scores obtained from each query.

*(iii) Average of multiple queries (MQ-Avg)* Similar to (ii), but the ranked lists are combined by scoring each retrieved image by the average of the individual scores obtained from each query.

In [1] we introduced two additional methods which use discriminative learning. However, these were found not to perform significantly differently to the other non-discriminative methods used here and are not compatible with the underlying Hamming embedding retrieval system (Sect. 2.3).

### Spatial reranking

Precision of a retrieval system can be improved by reranking images based on their spatial consistency [42, 47] with the query. Since spatial consistency estimation is computationally relatively costly, only a short list of top-ranked results is reranked. We use the spatial reranking method of Philbin et al.  [42] which reranks images based on the number of matching descriptors consistent with an affine transformation (inliers) between the query and the database image.

Here, we explain how to perform spatial reranking when multiple queries are used. For fair comparison of different methods, it is important to fix the total number of spatial transformation estimations and fix it to $R=200$ per image in the query set of size $N$.

For the *Joint-Concat* method which performs a single query, reranking is performed on the top $R$ results. Images are reranked based on the average number of inliers across images in the query set. The number of spatial transformation estimations is thus $N \times R$.

For methods *MQ-Max* and *MQ-Avg* which issue $N$ queries each, reranking is performed for each query independently before combining the retrieved lists. For a particular query (one of $N$), reranking is done on the top $R$ results using only the queried image. The number of spatial transformation estimations is thus, again, $N \times R$.

### Underlying single query image retrieval system

All methods in Sect. 2.1 make use of a standard single query retrieval system—*Joint-Concat* uses it to query with the concatenated query set, while *MQ-Max* and *MQ-Avg* use it to query with each query image independently. To this end, we implemented the Hamming embedding retrieval system [23] with burstiness normalization [24]. RootSIFT [2] descriptors are extracted from the affine-Hessian interest points [35], quantized into 100k visual words, and a 64-bit Hamming embedding [23] signature is stored together with each feature to improve feature matching precision. Two features are deemed to match if they are assigned to the same visual word and their Hamming signatures are within a standard threshold of 24 on the Hamming distance [23, 51]. For a given query, a similarity score for a database image is obtained by summing all the Gaussian weighted votes of the image’s matching features (a standard parameter value of $\sigma =16$ is used, as in [24, 51]). Finally, burstiness normalization of [24] is applied as well. We follow the common practice [10, 22, 23, 43] of using an independent dataset, Paris 6k [43], for all training, i.e. computation of the visual vocabulary and Hamming embedding parameters.

Spatial reranking is performed on the top $R=200$ retrieved results using an affine transformation [42]. To alleviate quantization errors, multiple assignment [43] to three nearest visual words is performed, but so as not to increase memory requirements this is done on query features only, as in [25].

### Query set outlier removal

**Fig. 1:**
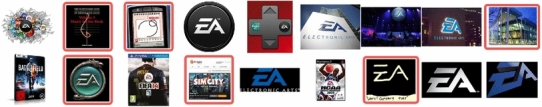
Automatic outlier removal. The top 18 images retrieved from textual Google Image search with the query ‘Electronic Arts’. Our automatic outlier removal procedure filters out all the outliers (shown with *red border*), while only making one mistake (false removal: *second row*, *second from left*). It is important to note that the visual diversity is preserved and surviving images contain: dark EA on bright background, bright EA on dark background, small-resolution EA logos (*second row*, fourth image, the *bottom right corner* contains the small logo), etc

The methods discussed thus far assume that the query image set is outlier free and tries to retrieve all database images relevant to all images in the query set. However, it is often useful to be able to form query sets automatically by crawling images from the Internet, as will be shown in Sect. 5 which uses textual Google Image search for this task. Such query sets which are not manually curated by a user often contain outliers because of imperfect search results, as well as inherent ambiguity in textual queries. An example is shown in Fig. 1 where a textual search for the ‘Electronic Arts’ company retrieves many relevant images from Google Image search which contain the recognizable EA letters, but there are also many outliers, including music for the book ‘The electronic arts of sound and light’, a digital drawing, a building facade and a game made by Electronic Arts which does not contain their logo.

We propose performing automatic outlier removal of images in the query set based on visual information. A key design goal is to remove outliers while maintaining visual diversity, as visual diversity is required for large recall. To this end, we employ a relatively loose consistency check where an image is deemed to be an outlier only if it is not similar enough to any other image in the query set. Two images are deemed to be similar enough if they share at least four matching descriptors. A descriptor match is defined in the standard way for a retrieval system which uses Hamming embedding (Sect. 2.3), namely two descriptors match if they are assigned to the same visual word and their Hamming signatures are within a threshold on the Hamming distance; here, a tight threshold of 16 (for 64-bit signatures) is employed. The procedure is motivated by the work of Tolias and Jégou [51] who demonstrate that accurate descriptor matching is sufficient for selection of reliable images for query expansion, without the need for using any geometric information traditionally required for query expansion [10, 11]. Figure 1 shows results of the automatic removal of outlier images in the query set.

Finally, outlier removal can be done very efficiently as it only requires pairwise Hamming distances to be computed between descriptors in the query set, and Hamming distance computation can be performed very fast on modern CPUs. Furthermore, the distances need to be computed only between descriptors assigned to the same visual word. While easily parallelizable, the entire procedure only takes 4 ms on average using a single thread.

### Evaluation and results

In this section we assess the retrieval performance of our multiple query methods by comparing them to a standard single query system, and compare them to each other.

#### Dataset and evaluation procedure

The retrieval performance of the proposed methods is evaluated using standard and publicly available Oxford Buildings [42] visual object retrieval benchmark. This dataset contains 5062 high-resolution images automatically downloaded from Flickr. It defines 55 queries (consisting of an image and query region of interest) used for evaluation (5 for each of the 11 chosen Oxford landmarks) and it is quite challenging due to substantial variations in scale, viewpoint and lighting conditions. The basic dataset, often referred to as *Oxford 5k*, is usually appended with another 100k Flickr images to test large-scale retrieval, thus forming the *Oxford 105k* dataset. Retrieval performance is measured in terms of mean average precision (mAP).

The standard evaluation protocol needs to be modified for our task as it was originally set up to evaluate single-query methods. We perform 11 queries, 1 per each predefined landmark; the performance is still measured using mAP.

Our methods are evaluated in two modes of operation depending on the source of the query set: one using the five predefined queries per landmark (Oxford queries, OQ), and the other using the top eight Google Image search results for the landmark names (Google queries, GQ), chosen by the user to make sure the images contain the object of interest. The images in the Oxford Building dataset were obtained by crawling Flickr, so we append a ‘-flickr’ flag to the textual Google Image search to avoid downloading exactly the images from the Oxford dataset which would artificially boost our performance. Note that the region of interest (ROI) is not provided for the GQ case, which makes this task more challenging.

#### Baselines

We compare our methods which use multiple query images to those that use a single image to query. For the Oxford queries (OQ) case the queries are the 55 predefined ones for the dataset, while for the Google queries (GQ) case thses are the 88 images downloaded from Google Image search. The two proposed baselines use exactly the same descriptors and vocabulary as our multiple query methods.

*Single query* A natural baseline to compare to is the system of Jégou et al. as described in [24] with extensions of Sect. 2.3. The AP for each of the 11 query landmarks is computed as the average AP across single queries for that landmark.

*Single query oracle (‘cheating’)* The *single query* method is used to rank images using each query from the query set (the same query sets are used as for our multiple query methods) and the best performing query is kept. This method cannot be used in a real-world system as it requires an oracle (i.e. looks up ground truth).

### Results and discussion

**Fig. 2:**
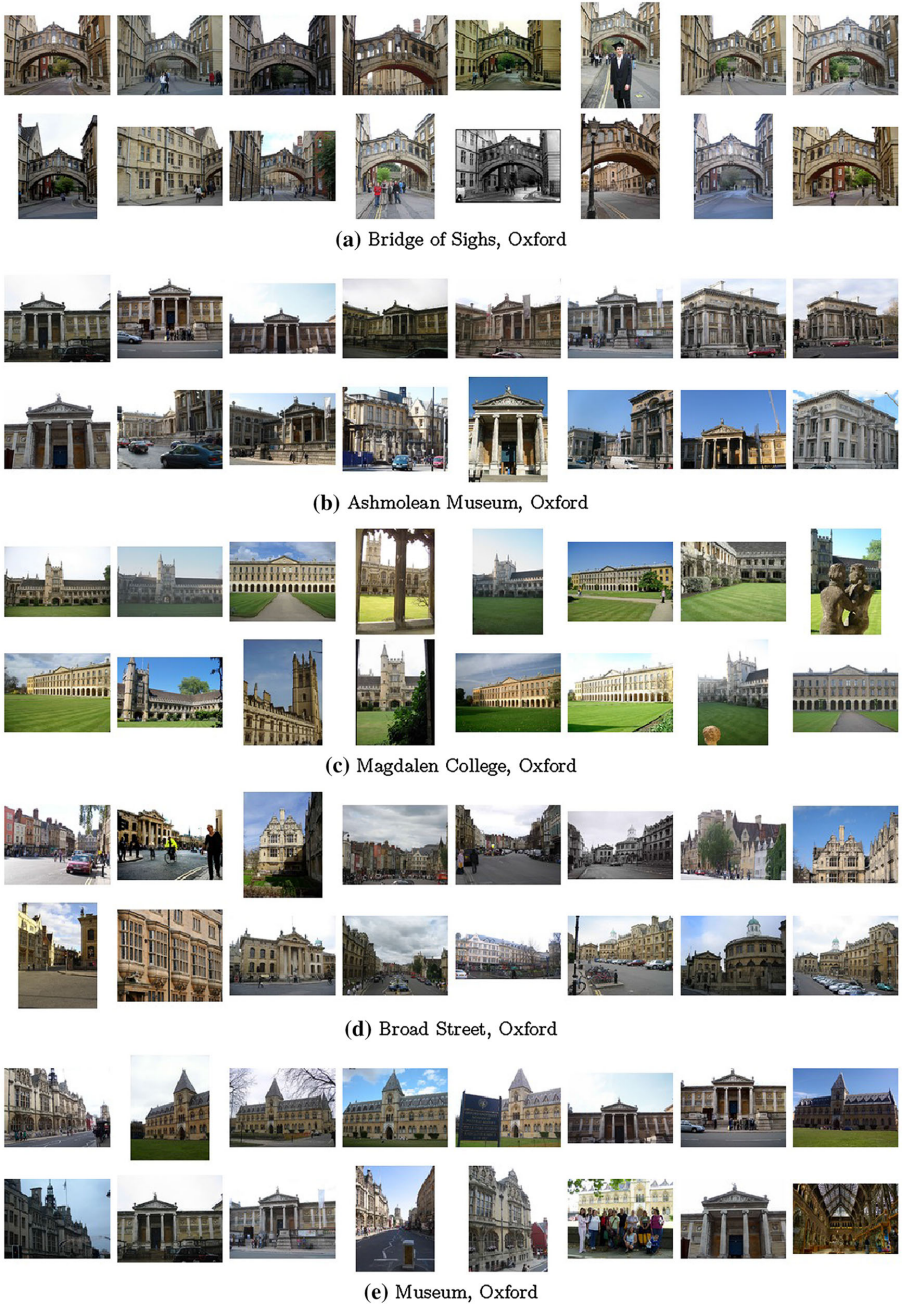
Query terms and top retrieved images from the Oxford 5k dataset. The captions show the textual queries used to download images from Google to form the query set. The top 20 images were used, without any user feedback to select the relevant one; the results are generated with the *MQ-Max* method. Specific (**a**, **b**) and broad (**c**, **e**) queries are automatically handled without special considerations. **e** Searching for ‘Museum, Oxford’, which is a broader query than **b**, yields in the top 16 results photos of three Oxford museums and a photo from the interior of one of them

Figure 2 shows a few examples of textual queries and the retrieved results. Note the ability of the system to retrieve specific objects (e.g. the Ashmolean museum in Fig. 2b) as well as sets of relevant objects (e.g. different Oxford museums in Fig. 2e) without explicitly determining the specific/general mode of operation.

Table 1 shows the retrieval performance on the Oxford 105k dataset. It can be seen that all the multiple query methods are superior to the ‘single query’ baseline, improving the performance by 41 and 78 % for the Oxford queries and Google queries (without spatial reranking or multiple assignment), respectively. It is clear that using multiple queries is indeed very beneficial, as the best performance using Oxford queries (0.891) is better than the best reported result using a single query (0.850 achieved by [50]. The method uses query expansion, so in a sense it does make use of multiple images); it is even on par with the state of the art on a much easier Oxford 5k dataset ([51]: 0.894). All the multiple query methods also beat the ‘single query oracle’ method which uses ground truth to determine which one of the images from the query set is best to be used to issue a single query.

**Table 1:** Retrieval performance (mAP) of the proposed instance search methods on the Oxford 105k dataset

	Google queries (GQ)				Oxford queries (OQ)			
		SR	MA	MA + SR		SR	MA	MA + SR
Single query	0.433	0.479	0.492	0.524	0.616	0.665	0.682	0.729
Single query oracle	0.733	0.762	0.779	0.800	0.754	0.816	0.814	0.849
Joint-Concat	0.789	0.798	0.788	0.812	0.863	0.878	0.874	0.887
MQ-Max	0.664	0.778	0.707	0.801	0.814	0.861	0.843	0.881
MQ-Avg	0.765	0.796	0.783	0.821	0.868	0.880	0.876	0.891

SR and MA stand for spatial reranking and multiple assignment, respectively. The source of the query images is either five predefined images (‘Oxford queries’) images, or the top eight Google images which contain the queried object (‘Google queries’). Details of the evaluation procedure, baselines and proposed methods are given in Sects. 2.5.1, 2.5.2 and 2.1, respectively. All proposed methods significantly outperform the ‘single query’ and ‘single query oracle’ baselines

**Fig. 3:**
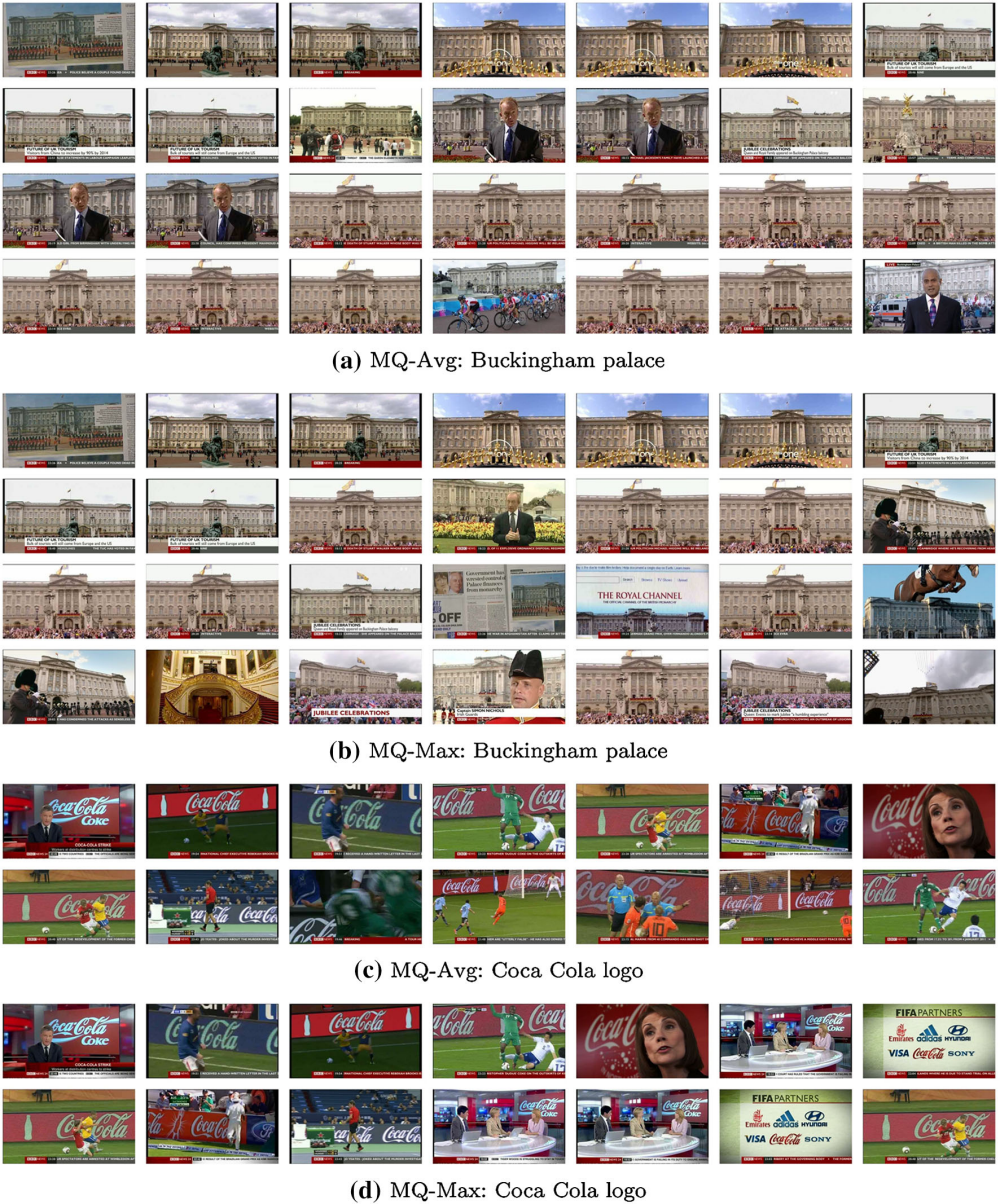
Multiple query instance retrieval on BBC News dataset. **a**–**d** Two different textual queries and retrieval results of *MQ-Avg* and *MQ-Max* methods. The *Joint-Concat* method is omitted for space reasons, but its behaviour is quite similar to *MQ-Avg*

From the quantitative evaluation it is clear that multiple query methods are very beneficial for achieving higher recall of images containing the queried object; however, it is not yet clear which of the three proposed methods should be used, as all of them perform very well on the Oxford 105k benchmark. Thus, we next analyse the performance of various methods qualitatively on the BBC News dataset (introduced in Sect. 1) and show two representative queries and their outputs in Fig. 3.

The *MQ-Max* method clearly retrieves more diverse results than *MQ-Avg* and *Joint-Concat* (due to lack of space we do not show *Joint-Concat*, but its behaviour is similar to *MQ-Avg*)—this is because taking the maximal score of the retrieved lists enables it to rank an image highly based on a strong match with a single query image from the query set. The other two methods which average the scores downweigh potential challenging examples even if they match very well with one query image, thus only retrieving ‘canonical’ views of an object. For example, while *MQ-Avg* retrieves mostly frontal views of Buckingham Palace (Fig. 3b), *MQ-Max* manages to find a few more challenging images (Fig. 3a): an image from a newspaper, more side views, as well as one photo from its interior. Similarly, *MQ-Avg* finds Coca Cola logos mostly coming from ads in football games (Fig. 3c), while *MQ-Max* discovers extra images where the logo appears in a news studio and on a list of FIFA partners (Fig. 3d).

It is also interesting to compare *MQ-Avg* with *Joint-Concat* to understand whether it is better to issue multiple queries and then merge the resulting ranked lists (the *MQ-*approaches), or to have a joint representation of the query set and perform a single query (*Joint-Avg*). In our qualitative investigations, we observed that the ‘multiple queries’ approach performed better. The argument for this is similar to those made in favour of the *MQ-Max* method, namely that it is beneficial to be able to find close matches to each individual query image. Furthermore, we believe that the spatial reranking procedure (Sect. 2.2) of the *MQ-*methods is more efficient—estimation of a spatial transformation between a query image and a short list is conducted on the short list obtained from the corresponding query image, while for *Joint-Concat*, where only a single ‘global’ short list is available, many attempts at spatial verification are wasted on using irrelevant query images. Another positive aspect of the ‘multiple queries’ methods is that they can be parallelized very easily—each query is independent and can be handled in a separate parallel thread.

Finally, taking all aspects into consideration, we conclude that the method of choice for multiple query retrieval is *MQ-Max*, where each image from the query set is queried on independently and max-pooling is applied to the retrieved sets of results.

## Object category retrieval

In contrast to the instance retrieval modality described in the previous section, which excels at finding specific objects (e.g. find all images of Westminster Cathedral), the object category modality is designed to handle queries of a broader nature (e.g. find images of *all* cathedrals).

**Fig. 4:**
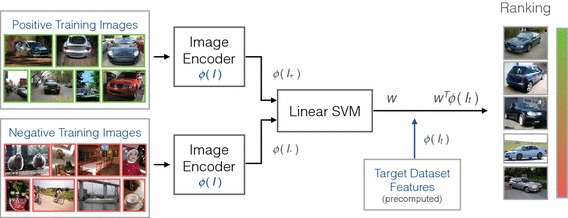
Architecture of a typical object category retrieval pipeline. Positive and negative training images can be either sourced from a separate training split of the target dataset, or from some other source such as Google Image search

The structure of a typical object category retrieval pipeline is illustrated in Fig. 4. In contrast to the instance retrieval setting, a learning stage is introduced in the form of a support vector machine. By training the SVM with a selection of positive training images that sufficiently capture both the commonalities and differences of appearance that occur within a class, more general queries are possible than with the instance retrieval modality.

### Visual features

Perhaps, the single most important design choice in such a pipeline is the selection of the image encoding function $\phi (I)$. We build on research that shows that deep ConvNet features significantly outperform shallow features, such as Fisher vectors [6, 41], on the image classification task [7, 28, 53].

As shown in [13, 53], the vector of activities $\phi _{\mathrm {CNN}}(I)$ of the penultimate layer of a deep CNN, learnt on a large dataset such as ImageNet [12], can be used as a powerful image descriptor applicable to other datasets. We used code based on the open source Caffe framework [27] to pre-train our CNN model, using the settings described for the CNN-M network in Chatfield et al. [7].

Aside from providing state-of-the-art retrieval performance, one advantage that ConvNet-based features have over other alternative representations is that they are very compact. Furthermore, Chatfield et al. [7] have shown that the underlying network can be retrained to output features as low as 128-D, all without compromising significantly on retrieval performance (a drop of only $\sim$2 % is observed). We therefore similarly set the dimensionality of our feature layer to 128-D.

*Compression* Optionally, these already very compact codes can be compressed further using binary compression methods. We explore the use of *product quantization (PQ)*, which has been widely used as a compression method for image features [26, 44] and works by splitting the original feature into $Q$-dimensional sub-blocks, each of which is encoded using a separate vocabulary of cluster centres pre-learned from a training set. We explore compression using $Q=4,8$-dimensional sub-blocks.

### Experiments and evaluation protocol

We quantitatively evaluate the retrieval performance of the system using two datasets:

*PASCAL VOC 2007* [15] comprises around 10,000 images downloaded from the photo-sharing site Flickr. We use the provided train and validation splits for training, and the test split for testing. Full annotation is provided for 20 different object classes within those images, and we use these classes as the basis of our evaluation. This dataset is used to provide a baseline evaluation of our system across a standard and widely used object category retrieval benchmark.

*MIRFLICKR-1M* [19, 20] is a much larger dataset, comprising 1M unannotated images (aside from quite noisy image tags). The dataset represents a snapshot of images taken by popularity also from the image-sharing site Flickr, and thus is more representative of typical Web-based consumer photography than ImageNet [12], which although also sourced from Flickr was collected through queries for often very specific terms from WordNet. This dataset is used to provide an evaluation of our system in a more realistic large-scale setting.

Finally, we evaluate the performance of our methods qualitatively over the BBC News dataset described in Sect. 1.

#### Evaluation protocol

In all cases, we are interested in evaluating the performance within an object category retrieval setting, and so measuring the ‘goodness’ of the first few pages of retrieved results is critical. We therefore evaluate using precision at $K$, where $K=100$, on the basis that the larger the proportion of true positives for a given object category at the top of a ranked list, the better is the perceived performance.

Adopting such an evaluation protocol also has the advantage that we are able to use the 1M images from the MIRFLICKR-1M dataset despite the fact that full annotations are not provided. Since we only need to consider the top $K$ of the ranked list for each class during evaluation, we can take a ‘lazy’ approach to annotating the MIRFLICKR-1M dataset, annotating instances of each PASCAL VOC class only as far down the ranked list as necessary to generate a complete annotation for the top-$K$ results. This avoids having to generate a full set of annotation for all 1M images.

We investigate two training scenarios. In the first we use the PASCAL VOC classes and the training split from the PASCAL VOC 2007 dataset to train our classifier. Secondly, we switch over to the use of training data from Google Image search. This is to test how the system responds in a more realistic retrieval scenario, where training data is sourced on-the-fly. A fixed pool of $\sim$16,000 negative training images is sourced from the Web by issuing queries for a set of fixed ‘negative’ query terms^1^ to both Google and Bing Image search, and attempting to download the first 1000 results in each case, and for each class around $\sim$1000 positive training images are retrieved.

### Results and analysis

The results of our experiments over both VOC 2007 and the MIRFLICKR-1M dataset are presented in Table 2. We first explore the performance of the pipeline using the VOC train split for training and the VOC test split for evaluation. This provides a good baseline of the performance of our features on a standard retrieval benchmark, and the features perform excellently with even the worst performing classes (‘cow’ and ‘bottle’) yielding 88 % precision at 100, and 13 out of the 20 classes performing at a perfect 100 % precision at 100.

**Table 2:** Object category retrieval results (Mean Prec @ 100) over the PASCAL VOC 2007 and MIRFLICKR-1M datasets

		VOC training		Google training	Storage/1M ims. (MB)	Comp. time/im (s)
		VOC 2007	MIRFLICKR	MIRFLICKR		
(a) CNN	128	92.1	95.1	92.3	488	0.34 (0.061)
(b) CNN	128 PQ	90.1	94.6	92.1	30.5	+3.9 ms
(c) CNN	128 PQ-8	88.8	93.1	91.1	15.3	+2.0 ms

*PQ* and *PQ-8* indicate the application of product quantization to compress the codes, using 4-dimension and 8-dimension subquantizers, respectively. Storage and computation time for each representation are also given

Switching to the MIRFLICKR-1M dataset, but still using the VOC training data, actually results in a slight improvement of the performance across the board. This scenario provides a closer representation of the performance of a real-world on-the-fly object category retrieval system, given that the image statistics of the MIRFLICKR-1M dataset are not known in advance, and the good performance indicates that the pipeline is able to scale to a larger, uncurated dataset, with the greater diversity of images that is implied.

Finally, switching over to the use of training data from Google Image search in place of the VOC training split and again evaluating over the MIRFLICKR-1M dataset as expected result in a small drop in performance ($\sim$2–3 %) for all methods. Nonetheless, the average precision at 100 in all cases remains above 90 %, indicating that despite cross-domain issues the training data from Google Image search combined with our CNN-based visual features are descriptive enough to tackle all of the PASCAL VOC classes.

**Fig. 5:**
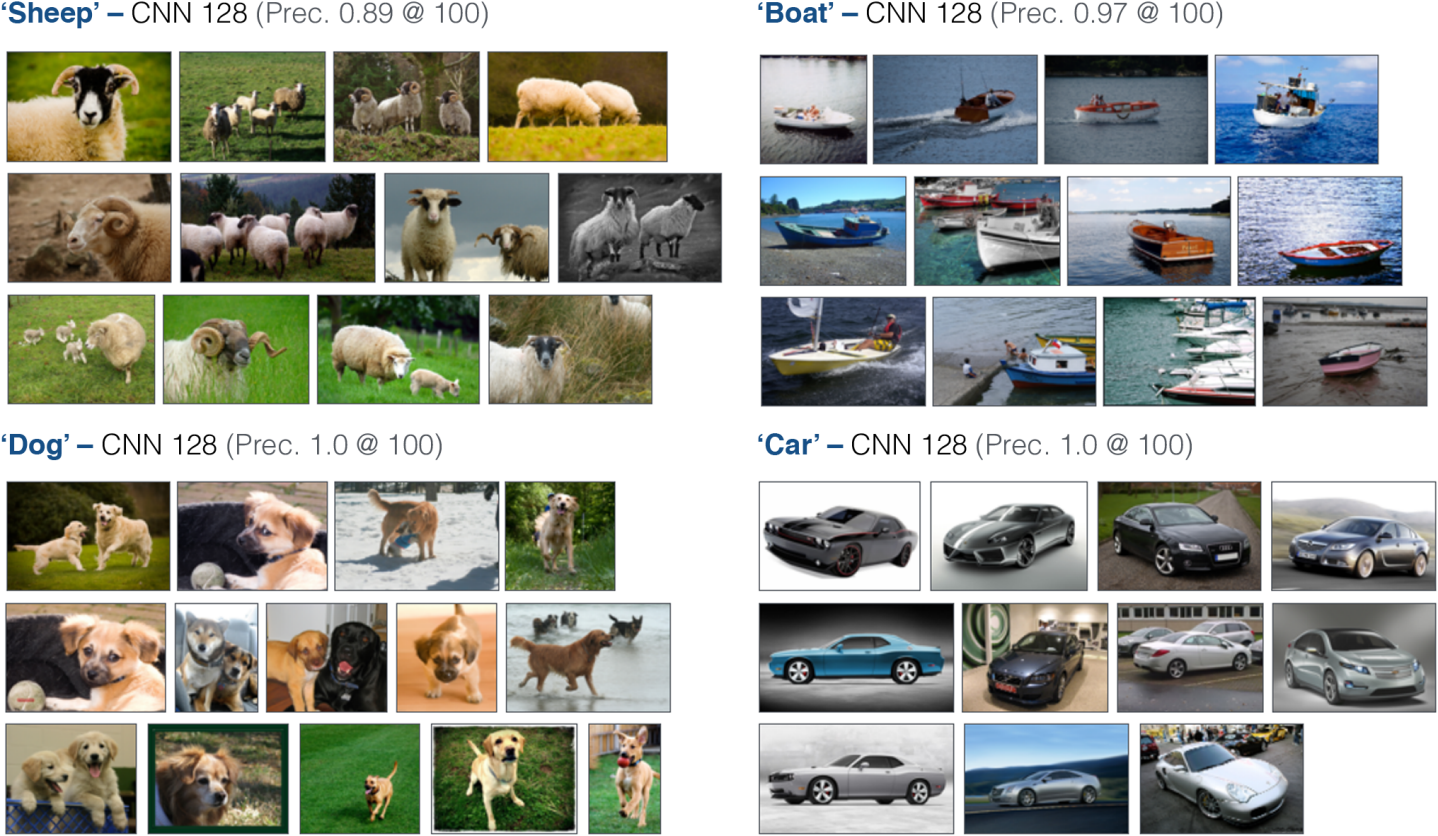
Object category ranking results over the MIRFLICKR-1M dataset (queries within the PASCAL VOC classes)

**Fig. 6:**
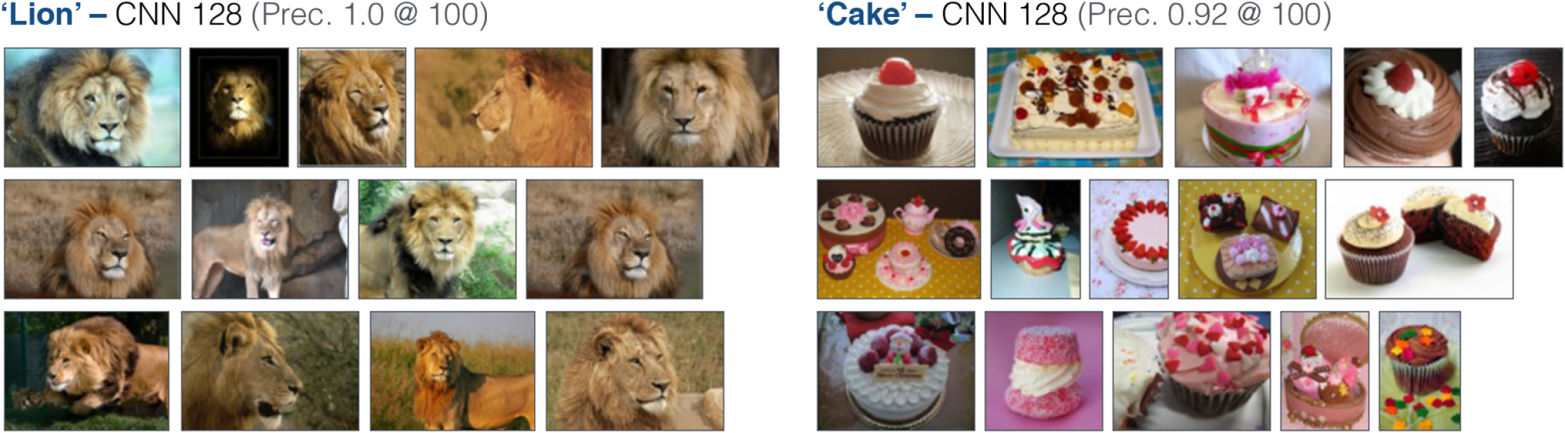
Object category ranking results over the MIRFLICKR-1M dataset (queries outside the PASCAL VOC classes)

Some sample ranking results for some of the VOC classes are shown in Fig. 5 using training images from Google Image search. However, as mentioned earlier the advantage of an on-the-fly architecture is that no limitation is imposed on the object categories which can be queried for, as a new classifier can be trained on demand. We present sample ranking results for some query terms disjunct from the 20 PASCAL VOC classes in Fig. 6 to demonstrate that the architecture is very much generalizable to query terms outside of the PASCAL category hierarchy.

**Fig. 7:**
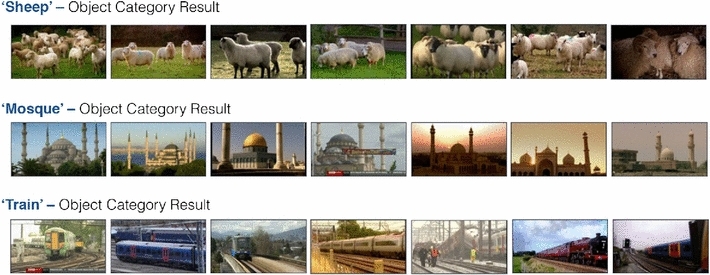
Object category retrieval results over the BBC News dataset using images from Google Image search for training

*Results over BBC News dataset* Some sample ranking results over the BBC News dataset, which is larger still than the MIRFLICKR-1M dataset by an order of magnitude, are shown in Fig. 7. It can be seen how even when applied over a large dataset from a very different domain, the approach scales well.

## Face retrieval

The aim of this modality is to retrieve a particular object class—faces. It can be used to handle queries such as find all images of person X, where X is a politician or actor for example. The approach is quite similar to that of object category retrieval in the previous section, in that discriminative classification is used with the architecture of Fig. 4, including positive and negative training data and a linear SVM classifier. The difference is that the fundamental unit of retrieval is a face track, and the process of obtaining and representing these tracks is an important part of the pipeline.

### Video processing

We pre-process all videos in our target dataset with the objective of detecting all faces within a shot and associating all detections of the same person into contiguous face tracks.

*Face detection and tracking* A face track is a temporal connection of detected faces of a single person. Faces are detected using the OpenCV frontal face detector and are linked together using KLT tracks as described in [14]. The facial landmark detector of [14] is used to detect nine facial landmark points. These points are used in the face representation and also for selecting the best face to represent the track. False-positive face tracks are removed based on the landmark detection confidence and the length of the track. Table 3 gives statistics on the number of detections and face tracks in the datasets used here.

**Table 3:** Face retrieval dataset details

Dataset	Hours	Faces	Tracks
Buffy	16.5	1,212,471	21,053
TRECVID 2012 INS	188	479,004	13,171
TRECVID 2013 INS	435	5,141,166	149,225
BBC News	10,132	6M	2.1M

Number of faces detected (faces) and face tracks (tracks) in different datasets used for evaluation

### Face representation

All of our face representations are feature vectors of fixed dimension which will be used in the learning stage. An important choice is whether a feature vector is computed for each face detection individually, or a single feature vector is used to represent the entire face track. For example, the patch intensity-based descriptor [14] or intensity gradient descriptor [39] produces one vector per frame in a track. Obtaining a score for a track is then both time consuming and memory intensive, as the complexity depends on the number of frames. An alternative is to select a single representative frame or to aggregate across the face track. The recently introduced Fisher vector-based descriptor [38] aggregates to produce a single feature vector for a face track. As described in Sect. 3.1, these features can be compressed using product quantization [26].

### Datasets and evaluation protocol

We use three datasets for the qualitative evaluation of our approach, the details of which are given in Table 3:

*Buffy* This is formed of 22 episodes of season 5 of ‘Buffy the Vampire Slayer’ and forms the core of our primary evaluation dataset. Different versions of this data have appeared in literature before [14, 46, 49]. We use the data publicly available at [3] that contains face tracks and associated ground truth for all 22 episodes. Ground truth annotations are provided for the tracks of the six primary characters in the series, namely Buffy, Willow, Xander, Giles, Tara and Anya. The first ten episodes provide training tracks for these characters and the rest of the episodes form the test split.

*TRECVID 2012 INS* This dataset was introduced for the TRECVID instance search competition in 2012. It consists of videos downloaded from Flickr under the creative commons licence. The face tracks of this dataset are used for negative training data in the experiments.

*TRECVID 2013 INS* This dataset was introduced as a part of TRECVID instance search competition in 2013. The dataset consists of about 214 episodes from the BBC television series ‘EastEnders’. The face tracks from the 78 episodes of this dataset are used as distracters in the experiments to make the task of recognition harder. Since the data are collected from a disjoint TV series, it makes it a perfect choice as a distractor for the the Buffy dataset.

For each character, the quality of the trained model is assessed using *Precision @ k*, i.e. the fraction of the top-k ranked results that are classified correctly and also the *average precision*. Finally, we further evaluate performance qualitatively over the BBC News dataset described in Sect. 1.

### Experiments

Our objective is to assess the quality of the retrieved face tracks for a given character. There are two specific goals: first, to find the best representation of a face track; second, to assess the suitability of Google Images for training character-specific models.

The training procedure is common to all the experiments described below. Given a character, a linear SVM classifier is trained using all tracks belonging to that character from the training data as positive examples, while tracks of all other characters and all tracks from TRECVID 2012 INS are used as negative training examples. The trained classifier is used to rank tracks from the test split of the Buffy dataset combined with the whole of the TRECVID 2013 INS dataset.

We compare four methods of representing and scoring face tracks. The first three are variants of the intensity gradient descriptor [39], whilst the fourth uses the Fisher vector face track representation [38]:

*LM* We select one representative frame of the track using the maximum facial landmark detector confidence score and represent the track by the feature vector of the face in the selected frame. The advantage of this method is that there is just one SVM score computation required per track.

*Max* Every face of the track is scored using the SVM, and the maximum score obtained is assigned to the track. Unlike the *LM* method, feature vectors for *all* faces of the track must be stored as they are required for classification.

*Avg* A single vector representation is computed for the track by averaging over all face descriptors. The track is then scored by the SVM of the average vector (due to linearity, this is equivalent to averaging the scores over all faces). As with the *LM* method, only a single feature vector needs to be stored per track.

*FV* A single Fisher vector is computed for the whole track.

Table 4 shows the performance of each of these methods over the combined Buffy + TRECVID 2013 INS dataset. For the intensity gradient descriptor, it can be seen that taking the maximum score for all frames in a track (*Max*) performs best. However, the Fisher vector representation outperforms all other methods, whilst only producing one feature per track in contrast to the *Max* method.

**Table 4:** Face retrieval experiments

Character	Train tracks	Test tracks	Grad. feat			FV
			LM	Max	Avg	
Buffy	2000	2179	0.960 (0.520)	0.909 (0.526)	0.970 (0.518)	0.970 (0.784)
Giles	504	630	0.960 (0.350)	1.000 (0.450)	0.818 (0.279)	1.000 (0.798)
Xander	795	841	0.990 (0.463)	0.990 (0.519)	0.960 (0.399)	1.000 (0.813)
Willow	720	1146	0.899 (0.311)	0.869 (0.361)	0.838 (0.255)	1.000 (0.748)
Tara	318	619	0.879 (0.273)	0.939 (0.342)	0.869 (0.230)	1.000 (0.697)
Anya	449	762	0.869 (0.251)	0.869 (0.285)	0.778 (0.204)	1.000 (0.715)
Mean	–	–	0.926 (0.361)	0.929 (0.414)	0.872 (0.314)	0.995 (0.759)

Prec @ 100 and average precision (in brackets) for different characters and experiments. Train and test track statistics are from the Buffy dataset. With the addition of distractors from the TV13INS data, the total number of test tracks is 124,761. All the performance figures are reported on this combined test set. The track representation experiments using gradient descriptors (Grad. Feats) show that selecting the maximum score (Max) amongst all detections from a track is the best strategy. Selecting one frame based on facial landmark score (LM) performs comparably in terms of Prec @ 100 while reducing memory and computational requirements

Table 5 compares the performance of a classifier trained on ground truth data to the one trained using images obtained from Google. For the characters where Gooogle can return both sufficient and accurate images to train a classifier, the results are comparable to the ground truth classifier, whilst performance drops in cases where Google cannot provide sufficient and accurate images for the character.

**Table 5:** Comparison of training data sources for face retieval

Character	Ground	Truth	Google	
	Grad	FV	Grad	FV
Buffy	0.960	0.970	0.626	0.677
Giles	0.960	1.000	0.162	0.182

Per character Prec @ 100 for classifiers trained on different data sources. The images available for a character from Google vary in quality. For the primary character of the series ‘Buffy’, Google returns sufficient images to train a classifier well. For a secondary character, such as ‘Giles’, the performance reduction observed is due to the lower quality of training images available from Google

**Fig. 8:**
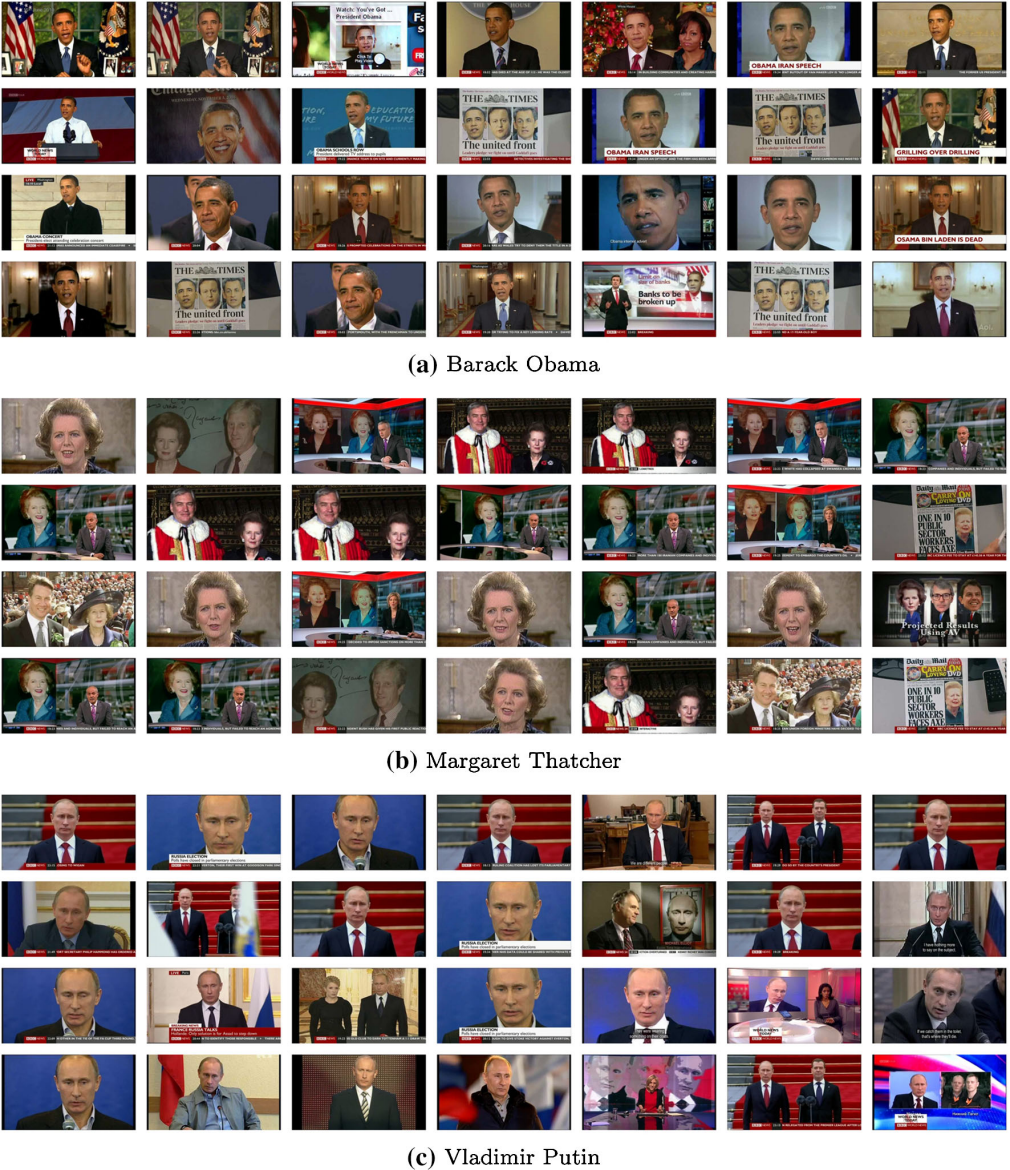
Face retrieval search examples. Top results for queries Barack Obama, Margaret Thatcher and Vladimir Putin on the BBC News dataset

*Results over the BBC News dataset* Figure 8 Example results of face retrieval for the BBC News dataset. In this case the training images are sourced from Google instead of from a curated dataset. As can be seen, we succeed in retrieving faces of a specific person with varying expressions and illumination.

## Building an on-the-fly system

This section describes the architecture of an on-the-fly system and how the three methods of the previous sections are implemented within it. As always, the issues are what to compute and store in advance vs. online and the memory–speed trade-off.

**Fig. 9:**
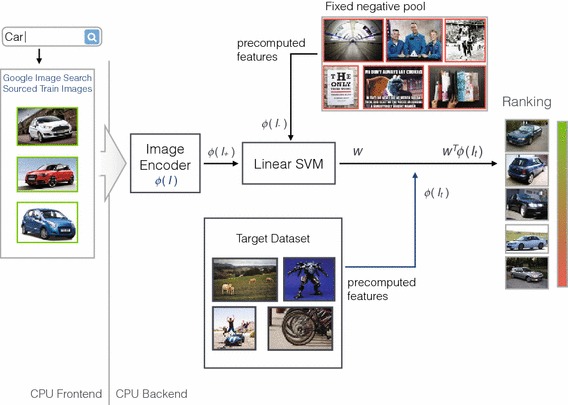
Architecture of the on-the-fly object category retrieval pipeline. Positive training images are downloaded on-the-fly from Google Image search by the front-end component and then fed to the back end for processing. In the back end, the images are encoded and after a fixed timeout of $\tau$ seconds all encoded images are fed to a Linear SVM for batch training (along with a fixed pool of negative features pre-computed during the pre-processing phase). Finally, the linear SVM model **w** is applied to the precomputed features of the target dataset and the resulting classification scores sorted in descending order to produce the output ranking

The on-the-fly system architecture for the case of object category retrieval (Sect. 3) is shown in Fig. 9 (compare to Fig. 4 for the non-on-the-fly version). We will describe this case first and then outline the differences required for instance or face retrieval.

The architecture is split into a pre-processing and online stage:

*Pre-processing* Visual features are extracted for all images in the target dataset, along with those for a fixed pool of $\sim$16,000 negative training images. The negative images are sourced from the Web by issuing queries for a set of fixed ‘negative’ query terms (in the same way as described in Sect. 3.2.1). Both the dataset features and those of the negative training pool are then stored in memory when the system is launched for speed of access.

*On-line stage* Given a textual query, the corresponding top $K\sim 100$ images are retrieved from Google Image search. Visual features are computed on-the-fly for each image as it is downloaded. These images are used as the positive visual training data for our object category. Along with the fixed pool of pre-computed negative training data, these are used to train a linear SVM $\langle \mathbf {w},\phi (I)\rangle$ by fitting $\mathbf {w}$ to the available training data by minimizing an objective function balancing a quadratic regularizer and the hinge loss. The parameter $C$ in the SVM is set to a constant value of $C=10$.

Having learnt a classifier for our concept, we then apply it to our dataset features, computed in the pre-processing phase. Following this, the output classification function is used as a ranking metric, and the images are presented to the user in descending order of these scores.

### System architecture

The architecture of the system can be broadly split into a single front-end and multiple back-end components.

*Front-end component* The front-end component is in charge of presenting the Web interface, managing requests and converting textual queries to visual training data by downloading images from Google Image search. It is written in Python.

*Back-end components* There is one back-end component for each search modality and each manages the process of producing a ranked list of results given a query. The back-end components are all written in C++ for speed and efficiency, as they are the most computationally expensive part of the system.

### Implementation details

*Obtaining positive training images* The image downloader component is implemented in Python using co-routines to maximize the rate at which training images can be downloaded. Our target is to download between 20 and 150 training images, depending on the search modality. We do this by first requesting the first 300 results from Google and then impose a timeout of $\tau _\mathrm{image}\sim 100$ ms on the download time of each image, which ensures that no undue time is wasted retrieving images from slow servers. A global timeout is also set between $\tau _\mathrm{global}\sim 1$ and 5 s depending on how many training images are required. Features are then computed in real time and in parallel over multiple CPU cores and stored in memory for training.

*Instance search* We have found it sufficient to use a query set of 20 images downloaded from Google for the instance search modality. For methods which issue multiple queries (*MQ-*), each query is executed in an independent thread and then merged.

*Category search* We use between 100 and 150 images from Google for the category search modality. When querying Google Image search, we ask it to only return ‘photos’, avoiding the pollution of the training data by line drawings and cartoon images. Ranking is conducted using a parallel approach which splits the target dataset between multiple computation threads.

*Face search* We use between 100 and 150 images from Google for the face search modality. When querying Google Image search, we ask it to only return ‘faces’. Again, ranking uses a parallel approach with multiple computation threads over the target dataset tracks.

### Memory requirements

The details of the BBC News dataset, along with the memory requirements of each search modality, are summarized in Table 6.

**Table 6:** BBC News dataset statistics and memory requirements

Dataset details	
No. of programmes	17,401
Hours	10,132
Keyframes	5.3M
Face tracks	2.1M
Memory req.	
Instances	21 GB
Categories	2.7 GB
Faces	32 GB
Categories (with PQ)	0.08 GB
Faces (with PQ)	2 GB

The BBC News dataset is sourced from all BBC News footage broadcast from 6 pm until midnight from 2007 to 2012 and is used for qualitative evaluation and the on-the-fly system. In the case of product quantized (PQ) figures for the category and faces modality, subquantizers of size 8 and 4 dimensions, respectively, are used

*Instance search* Posting lists in the inverted index are compressed by encoding differences of sorted image identifiers using variable-byte coding [54]. Furthermore, local affine shape of the affine-Hessian interest points (Sect. 2.3) are compressed using [40]. After compression, the instance search modality requires 42 GB of RAM for the entire BBC News dataset. To further reduce the memory requirements we subsample the dataset by two by removing every other frame from the index, and therefore halving the RAM usage to 21 GB.

*Category search* The ConvNet-based features used for category search are already very compact (128-D) and so we store and use them over the target dataset in uncompressed form. However, further compression can be achieved of up to 32$\times$ using product quantization as described in Sect. 3.1 and shown in Table 2.

*Face search* Using the frame selection strategy based on facial landmark detection (Sect. 4) significantly reduces memory requirements compared to processing all frames. However, as with the category search features, a further 16$\times$ compression can be achieved using product quantization.

### Web-based demo system

**Fig. 10:**
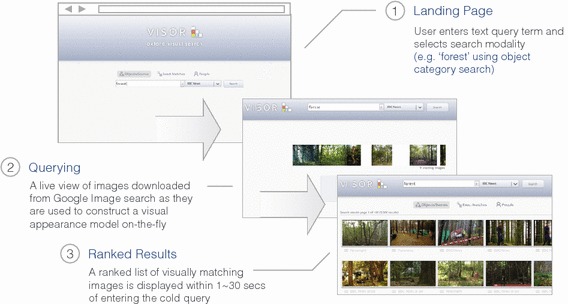
Web-based on-the-fly demo system. (*1*) the user enters a text query term and selects a search modality, (*2*) images are downloaded from Google Image search and used to train an appearance model on-the-fly, (*3*) ranked results over the target dataset are returned. A live demo is available online—see the text for details

An online demonstration of the three methods is available at http://www.robots.ox.ac.uk/~vgg/research/on-the-fly/ and illustrated in Fig. 10.

## Applications and extensions

In this section we overview a number of applications and comparisons of the approaches.

**Fig. 11:**
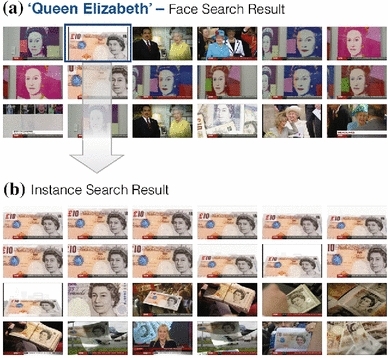
Interaction between face and instance search. Users can effortlessly switch between search modalities. In this example, querying for the British queen is done via face search (**a**), and the second result depicting the 10 pound note is used to issue an instance search query (**b**). Results are on the BBC News dataset

*Interaction between search modalities* An image or video database can be browsed intuitively by switching between search modalities—users can pick out results of previous searches to query the database potentially using a different modality. An example is shown in Fig. 11 where a user transitions from face to instance search.

**Fig. 12:**
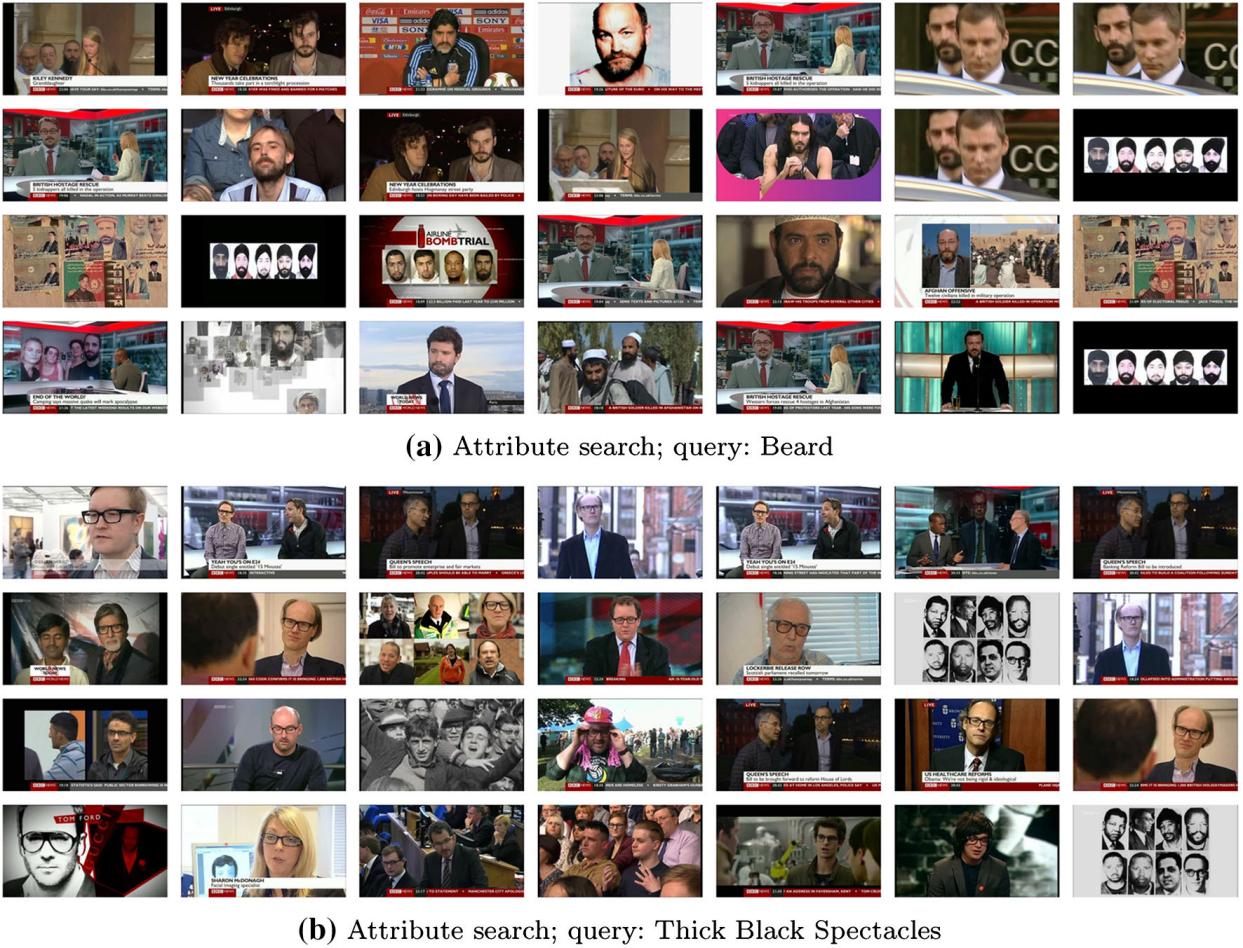
Face retrieval attribute search examples. Top search results for facial attribute queries ‘beard’ and ‘black spectacles’, respectively, on the BBC News dataset

*Facial attribute search* On-the-fly face classification can also be used for retrieving face tracks with specific attributes such as a moustache, beard, glasses and gender, by simply using these for the text query, rather than specifying a person (by name) as in the case of identity retrieval. This simple technique enables users to explore the content along other dimensions. Figure 12 shows several facial attribute examples on the BBC News dataset.

*Uber classifiers* The on-the-fly paradigm can be complemented by persistent classifiers learnt from curated datasets. These are termed ‘uber’ classifiers. The need for such classifiers is twofold: first, classifiers trained on-the-fly are subject to changes in the results of the image search engines—content on the Internet can change rapidly, e.g. for trending topics, and also slowly, e.g. with returns dominated by more recent (and thus older) faces of a particular actor or politician. Second, training classifiers off-line avoids the compromise between speed and accuracy, so far as larger training sets can be employed. For example, if the goal is to retrieve all occurrences of a politician in a static (archive) dataset, then using recent photos from a Web search may perform poorly, but a classifier trained on a corpus of images spanning several decades would likely perform better.

**Fig. 13:**
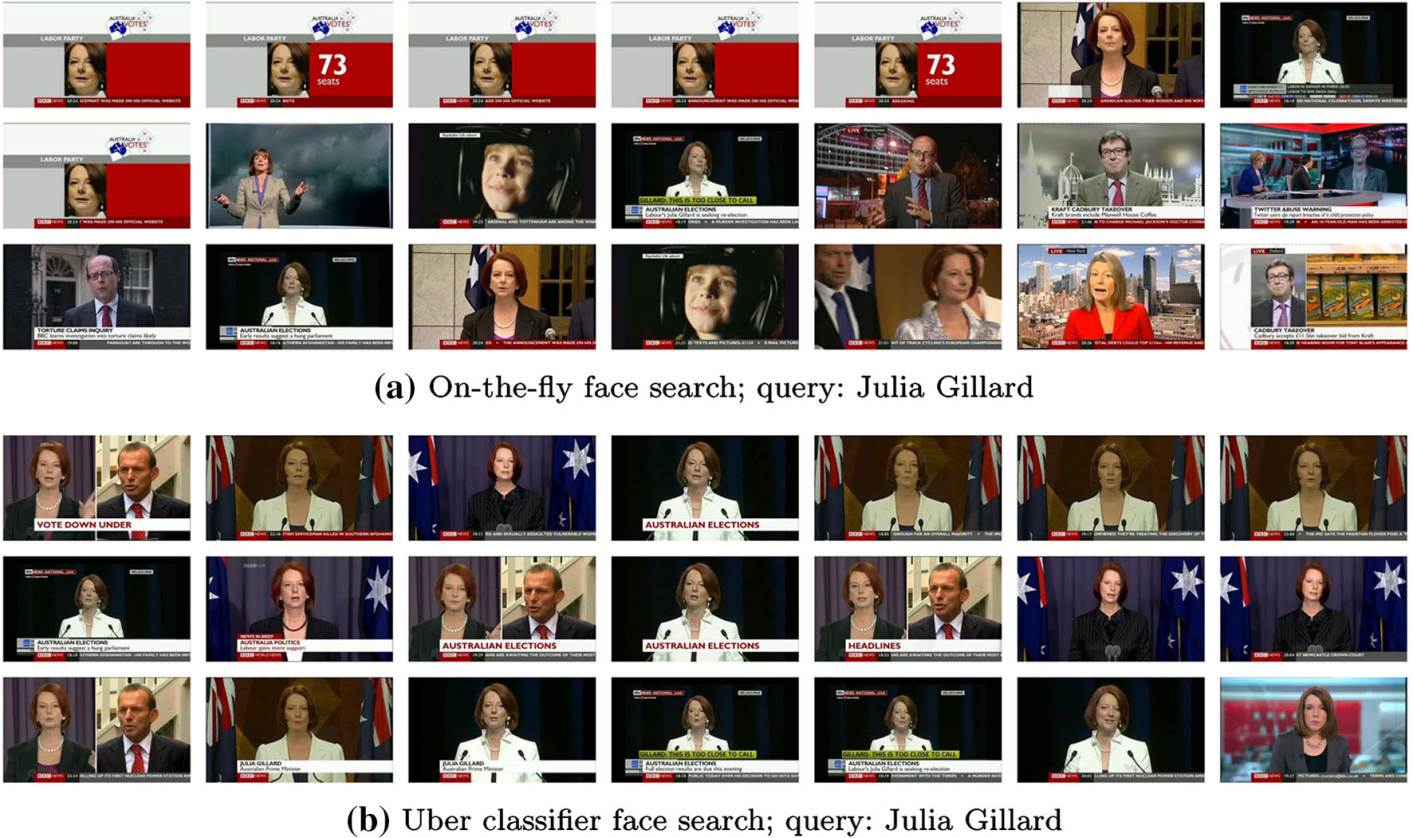
Face uber classifier example. The top search results when queried for the former Australian prime minister Julia Gillard. On-the-fly search results (*top row*) are quite poor, while the results with an uber classifier (*bottom row*) trained on curated data are much better. Results are on the BBC News dataset

Figure 13 One such example. Searching for the former Australian prime minister a few years after her tenure does not result in good results because of the poor training data available from the Web. However, after manually curating images from relevant websites, the uber classifier yields superior results.

**Fig. 14:**
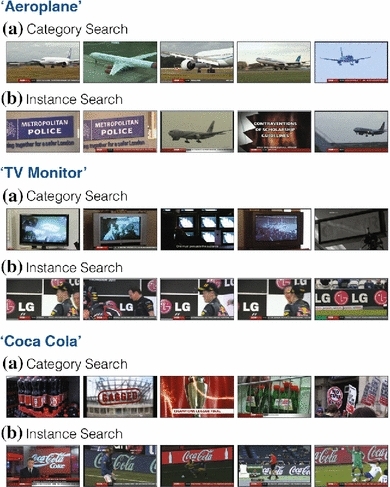
Comparison of category and instance search. The top five retrievals for the category and instance search modalities for the same three queries: ‘aeroplane’, ‘TV monitor’ and ‘Coca Cola’. The results are on the BBC News dataset

*Comparing different approaches for the same query* It is interesting to consider how category and instance searches perform against each other when faced with the same textual query; Fig. 14 shows three examples. As expected, category search works well for category queries ‘aeroplane’ and ‘TV monitor’, while instance search is superior for ‘Coca Cola’. However, the failure modes of both modalities are interesting to analyse. Category search fails for specific object queries by over-generalizing, for the ‘Coca Cola’ query it finds several bottles, not necessarily Coca Cola bottles (note that the bottles in the top retrieval obtained from category search are not of ‘Coca Cola’ but just of ‘Cola’). Conversely, instance search focuses on specific instances, for the ‘aeroplane’ query it manages to find a few aeroplane models which exist in the query set. The query ‘TV monitor’ also shows over-specialization of instance search where the results are polluted with LG logos.

## Conclusions and future work

In this paper we have illustrated the on-the-fly approach for three classes of queries: object instances, object categories and faces. The question, then, is what next? Can human pose be learnt on-the-fly from still images downloaded by search engines or human actions [21]?

The visual search competencies described here are each based on a different underlying technology (Bag of Visual Words, Image Classification, Face-track classification)—though as illustrated in Sect. 6 a particular query class can be handled by more than one underlying technology to some extent. This raises the question of how to choose which technology to use for a particular text query or, alternatively, how to combine the results if the text query is issued to all three search methods.

The two competencies that use discriminative learning (categories and faces) require a set of negative images for training. Presently, we use a fixed generic set of negatives for all queries. However, it is likely that a more sophisticated method which selects a set of negative images per query, according to their discriminativeness for the query at hand, would result in a further improvement in results. This could be via topic modelling within a larger pool of uniformly distributed dataset images, followed by the subsampling of ‘negative’ topics close to the decision plane. Alternatively, the use of an iterative negative ensemble learning approach using support vector machines as in [32] could be explored.

There are also a number of standard competencies that are provided by modern search engines: such as composite queries (e.g. ‘Obama standing outside the White House’), diversity of the returned results and clustering of the returned results. Each of these have a different twist when vision, rather than text, is the primary search and representation method. Developing these competencies for visual search opens up new research directions.

## Footnotes

1 Miscellanea, random selection, photo random selection, random objects, random things, nothing in particular, photos of stuff, random photos, random stuff, things.
